# Strategies of Lymph Node Dissection During Sublobar Resection for Early-Stage Lung Cancer

**DOI:** 10.3389/fsurg.2021.725005

**Published:** 2021-09-23

**Authors:** Dominique Gossot, Alessio Vincenzo Mariolo, Marine Lefevre, Guillaume Boddaert, Emmanuel Brian, Madalina Grigoroiu, Nicolas Girard, Agathe Seguin-Givelet

**Affiliations:** ^1^Department of Thoracic Surgery, Curie-Montsouris Thorax Institute–Institut Mutualiste Montsouris, Paris, France; ^2^Department of Pathology, Institut Mutualiste Montsouris, Paris, France; ^3^Department of Oncology, Curie-Montsouris Thorax Institute–Institut Curie, Paris, France; ^4^Faculty of Medicine Simone Veil, Paris Saclay University, UVSQ, Versailles, France; ^5^Faculty of Medicine SMBH, Paris 13 University, Sorbonne Paris Cité, Bobigny, France

**Keywords:** lymph node dissection, lung cancer, sublobar resection, lymph node, non-small cell lung cancer, thoracoscopic surgery, minimally invasive surgery

## Introduction

The standard surgical treatment of NSCLC remains lobectomy combined with lymph node (LN) dissection (LND), even for early stage tumors (1). However, there is a growing interest in sublobar resections (SLR), even in patients who can tolerate a lobectomy, as reflected by the increasing number of publications referenced in PubMed (2) as well as changing practices. Thus, in our department, the average rate of SLR varies between 25 and 35% (3). Trials comparing lobectomy and RSL are underway (4–6) and the favorable results of the JCOG-0802 study has recently been announced (publication pending at the time of writing this article).

Until now, the published survival results of SLR have been less satisfactory than those of lobectomies. For several years, after the publication of the Lung Cancer Study Group comparing lobectomies and SLR, which showed poor results on local recurrence and survival, lobectomy has been considered the gold standard treatment (7). Subsequent cohort studies have confirmed this attitude. In particular, the SEER database study of over 14,000 patients with NSCLC demonstrated significantly better survival after lobectomy, regardless of tumor size (8).

Surgeons have until now considered lobectomy to be the standard treatment for NSCLC–apart from cases requiring pneumonectomy for location reasons–not only because of the above-mentioned findings, but also to apply the recommendations and with the consideration that a lobectomy provides satisfactory safety margins and allows removal of the lymphatic networks and intralobar nodes. Several years ago, the presence of invaded interlobar nodes led to the extension of the resection to pneumonectomy. The poor oncological benefit and the high morbidity and mortality of pneumonectomies have led to abandon this dogma.

However, many recent monocentric studies have demonstrated the non-inferiority, or even a slight superiority of SLR over lobectomies for early-stage NSCLC (9–13). Currently, more and more teams are performing SLR for selected early-stage tumors. Presumably, they apply, or should apply, the same oncological rules as for lobectomies, which have been clearly defined (14–16), i.e., performing a macroscopically complete resection with free margins and a systematic lymph node dissection, according to the guidelines (14). One of the questions is therefore why intersegmental LN dissection and analysis of the resection margins are not routinely performed during SLR for cancer. The latter point is not the subject of this article and we will focus here on lymph node dissection during SLR.

The reasons for the possible inferiority of SLR compared to lobectomy, at least in some cohort studies, are the following (and these reasons can be correlated): (1) some SLRs are actually wedge resections, (2) insufficient resection margins, (3) low number of nodes being examined, (4) absence of analysis of the so-called “adjacent” nodes and (5) non-practice of frozen section on margins and on the segmental nodes, which does not allow for extension to lobectomy during the procedure (Positivity nodes on final pathological examination rarely leads to a reoperation). All in all, as emphasized by P. Thomas, lymph node dissection might be the key point in performing a “radical segmentectomy” (17).

In this article, we will look at the four areas of concern and end-up with proposals.

## The Issue “Wedge or Anatomical Segmentectomy”

If one considers that an SLR for cancer must be anatomical, one question is whether SLRs in some studies are true anatomical segmentectomies or just mere wedge resections. Indeed, it is known that the local recurrence rate after wedge resection is high, for two reasons: the lack of LN dissection and the limited safety margin. In their comparative study, Handa et al. demonstrated the average surgical margin was significantly lower in the wedge group (1.0 vs. 1.5 cm in the segmentectomy group) (18). Margins are especially of concern in case of associated Spread Through the Air Space which is a factor of local recurrence and concerns 15% of the patients (19). In the historic study by Ginsberg et al. comparing SLR to lobectomies, wedge and segmentectomies were not differentiated (7). It seems this confusion is still present in several recent studies (20). For example, Kamel et al. studied the outcome of patients with NSCLC detected by a screening program. While 74% of the tumors were early stage, only 16% of the patients had an SLR, the majority of these (69%) being wedge resections (21). In a recent study comparing local recurrence after 1,354 lobectomies vs. 333 SLRs, the local recurrence rate was significantly higher in the SLR group. However, the latter were predominantly wedges (*n* = 285), making results questionable (20). The ongoing prospective CALGB 140 503 trial may suffer from the same bias since the two types of resection are mixed (4), unlike the JCOG 0802 trial (5). The fact that many SLRs for cancer are in fact wedges is related to the issue of LN dissection since, by definition, there is no LN dissection during a wedge. In the study by Subramanian et al. the average number of lymph nodes removed in the SLR group was only one (20). In the study by Handa et al. the number of nodes examined was 0, compared to 6 in the segmentectomy group (18).

**Figure 1:**
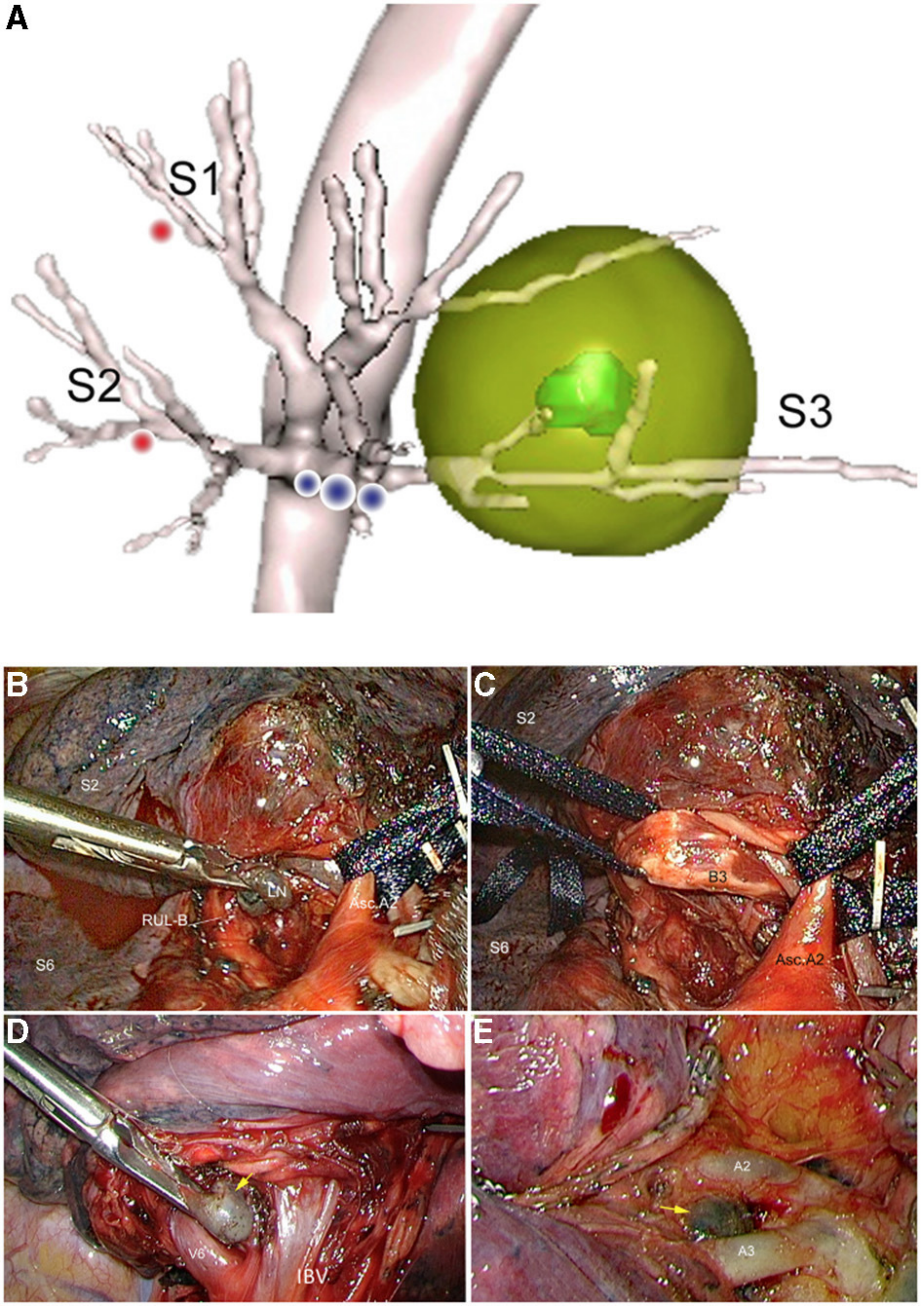
S^3^ Segmentectomy for a cT1aN0M0 NSCLC and examples of adjacent lymph nodes requiring intraoperative examination. **(A)** Schematic view based on 3D reconstruction (green mass = target nodule, pale yellow sphere = virtual safety margin, yellow dots = aLNs, red dots = iSLs). **(B)** Intraoperative view of aLNs hiding B^3^ bronchus, **(C)** Intraoperative view after LN clearance. *RUL-B, right upper lobe bronchus; LN, Lymph node; Asc.A2, ascending A*^2^ *artery*. **(D)** Suspicious LN adjacent to V^6^ during a planned right S^6^ segmentectomy. **(E)** Suspicious LN adjacent to A^3^b during a planned left S^2^ segmentectomy.

However, it has recently been shown that an anatomical segmentectomy has a better survival than a wedge in stage I tumors (18, 22). In addition, performing an anatomical segmentectomy theoretically requires the removal of peribronchial nodes, at least to ease dissection and division of the segmental bronchus (Figures 1A–C). The same applies to perivascular nodes (Figures 1D,E).

## The Influence of the Number of Nodes Removed on Survival

The number of dissected and retrieved LN during a major pulmonary resection can be debated as it varies according to the patient (e.g., some patients have few nodes), to the surgical procedure (depending if nodes were dissected en-bloc or fragmented) and to the pathological examination. However, in large series, the median number of LN is reliable. Node staging is a prognostic factor in NSCLC. It is accepted that a pN1 stage has a better prognosis than a pN2 stage. However, as with other cancers (breast, stomach, rectum), the number of metastatic nodes may be a more relevant indicator. Thus, in a series of 1,650 operated NSCLC, Wei et al. showed that 5-years overall survival was not significantly different between pN1 and pN2 (respectively 55.4 vs. 47.8%, *p* = 0.245) whereas there was a highly significant difference between nN0 (node-absent), nN1 (metastasis in one to two nodes), nN2 (metastasis in three to six nodes), and nN3 (metastasis in seven or more) (9.2, 65.1, 42.1, and 22.4%, respectively; *p* < 0.001) (23). Some retrospective analysis have suggested the possibility of minimizing (i.e., sampling) or even avoiding lymph node dissection in case of cT1aN0 NSCLC (in particular for pure GGO found during lung-cancer screening program) (24). However Darling et al. have shown that the number of mediastinal nodes removed had a favorable influence on prognosis by allowing more reliable staging and therefore a better indication of adjuvant treatments (25). According to other authors, the number of affected LN stations is also a prognostic factor, with 5-year survival decreasing from 72.1 to 58.3% and 29.6% depending on whether only one, two-three or four sites were affected (N1 and N2 sites combined) (26).

In 259 patients who underwent segmentectomy for NSCLC, Huang et al. showed that when patients had more than 6 nodes examined, the rate of diagnosed lymph node metastases was significantly higher (9.4 vs. 1.5%, *p* = 0.005) and 5-year survival was significantly higher (27). In Yendamuri's series of 3,916 patients operated on for stage I NSCLC by SLR, the only prognostic factor in multivariate analysis for 5-year survival was the number of nodes examined, while the extent of resection did not have a significant influence (28). The authors concluded that lymph node examination is a critical part of SLR.

## The Influence of the Location of The Segmental Nodes

For the following chapter, we will adopt the names of lobar and segmental nodes cited in Matsumura's article, i.e., *adjacent* (aLNs) for nodes located at the foot of the segmental bronchovascular elements of the resected segment and *isolated* for those located remotely in a bronchus or in the parenchyma of the remaining segments (iLNs) (29).

Some metastatic nodes in groups 13 and 14 are located deeply in the parenchyma and are undetectable both preoperatively and intraoperatively. A recent study showed that the probability of finding iLNs in cN0 patients was high. Of 196 patients operated on by lobectomy for stage I NSCLC, there were 36 pN1s and of these 36 pN1s, 28 were iLNs and 30 were aLNs (30). It is thus important to find indicators of the potential presence of these LN. At present, we only have an indirect indicator, i.e., the examination of adjacent nodes. In the above-mentioned study of Xiao et al. the probability of being iLNs positive was 40% when an aLN was invaded (30). The authors concluded that when an aLN was metastatic at frozen section, segmentectomy should not be recommended and resection should be extended to lobectomy.

## The Role of Intraoperative Examination

As SLRs are applied in the vast majority of cases to early-stage NSCLC, the finding of invaded segmental nodes is relatively rare. The problem is: should these nodes be positive, it would be too late if only discovered on the final pathological report. The question of an intraoperative examination is therefore relevant for patients having an SLR in an intention to treat.

In their series of 99 complex segmentectomies for early stage NSCLC, Handa et al. argue for frozen section which resulted in seven patients being converted to lobectomy, either for insufficient margin or for invaded segmental lymph node (12). We have reported the same experience with a switch to lobectomy for the same reasons in 5.1% of our patients (3). In the future, the development of sentinel node techniques, using either radioisotopes (31) or infrared imaging after systemic injection of indocyanine green, may help to perform intraoperative analysis only on a specific target (32).

Among arguments against frozen section of segmental node is that if the interlobar nodes were positive during lobectomy, the procedure would not be expanded into a pneumonectomy. This objection is true from an oncological point of view but false from a clinical standpoint as the consequences of an extension from lobectomy to pneumonectomy are nothing like those of an extension from segmentectomy to lobectomy, both in terms of morbidity and quality of life.

Another argument against intraoperative segmental lymph node analysis is the fact that frozen section would not be totally reliable. Its specificity is 100% but its sensitivity is only 85% (33). However, this is already much better than not doing it at all. Moreover, other pathology techniques, such as “touch preparation cytology” can improve sensitivity up to 95% (33). According to other authors, the traditional hematoxylin and esosin (HE) staining method used in frozen section may miss isolated tumor cells or micrometastases within the nodes analyzed (34). The authors suggest the use of rapid immunohistochemistry to obtain a reliable diagnosis within 20 min. Of 70 patients operated on for early-stage NSCLC by segmentectomy, five had segmental nodes positive using this method.

## Discussion

We have stressed the concern of insufficient LN clearance during SLR that impacts oncological survival in two ways: local recurrence by overlooking iLNs and mistaging by non-examination of aLNs. According to a recent study, “many patients having sublobar resection for early stage NSCLC in the United States do not have a single lymph node removed for pathologic examination” (28).

However, in addition to the aforementioned arguments, there is ample evidence in favor of an LN dissection.

Cox et al. showed that in a series of 1991 lipid adenocarcinomas that had either lobectomy or segmentectomy, survival was significantly better after lobectomy, except in the subgroup of SLRs associated with lymph node dissection. The authors concluded that lymph node dissection was essential during segmentectomy (35). These conclusions are in line with those of Wolf et al. and Matioli et al. in earlier publications (36, 37): segmentectomies have a survival equivalent to that of lobectomies only when lymph node dissection is performed.

From a technical standpoint, one of the questions is whether segmental LN dissection can be done adequately *via* closed chest surgery. It has been shown that dissection of hilar and peribronchial LN can be more difficult by VATS than by thoracotomy. Basing on a series of 11,500 cases of early-stage NSCLC, Boffa et al. have demonstrated that upstaging from N0 to N1 was more common in the open group (9.3 vs. 6.7%; *p* < 0.001) while upstaging from N0 to N2 was similar (5.0% open and 4.9% VATS; *p* = 0.52). The difference in the upstaging from N0 to N1 was however reduced after an average of 100 procedures (38). But, for surgeons performing closed-chest SLR, one may assume they are proficient and lymph node dissection is not a technical barrier since a very precise bronchial and vascular dissection must be performed anyway through a fissure- based approach. A difficulty sometimes emphasized is the time required for frozen section. However, as underlined by some authors, intraoperative analysis of aLNs takes little time and effort and should therefore be routinely done to assist in the decision of switching to lobectomy (30).

Meanwhile, many studies have reported the high pathological—complete or major—response after neoadjuvant treatment with immune checkpoint inhibitors; besides a potential shift in the treatment algorithms for early-stage neoadjuvant strategies, such finding may ultimately lead to raise the question of the actual need of extensive lung resections (39).

In conclusion, if SLR for early-stage NSCLC are performed with a curative intent, the same principles as for lobar resection should apply, that is radical resection with free margins and adequate LN clearance. One can even say that the level of exigency should be even higher because of the potential for overlooking iLNs. This leads us to make the following recommendations, which are not our own but those inspired by surgeons and expert centers that have been cited in this article (11, 17, 18, 23, 26, 27, 29, 40):

- Perform intersegmental node resection of the target bronchovascular pedicle and the remaining adjacent segment.
- Perform intraoperative examination of at least any suspected aLN and at best all aLNs, as invasion of any of these LNs may be a marker for metastatic iLNs.
- Switch to lobectomy in case of positive aLN.
- Complete with radical mediastinal lymph node dissection, as for any major pulmonary resection, because skip metastases are possible.

## Author Contributions

DG has contributed to conception design of the study and writing of the first draft of the manuscript. AM and AS-G have contributed to conception and design of the study. All authors contributed to manuscript revision, read, and approved the submitted version.

## Conflict of Interest

The authors declare that the research was conducted in the absence of any commercial or financial relationships that could be construed as a potential conflict of interest. The handling Editor declared a past co-authorship with one of the authors AM.

## Publisher's Note

All claims expressed in this article are solely those of the authors and do not necessarily represent those of their affiliated organizations, or those of the publisher, the editors and the reviewers. Any product that may be evaluated in this article, or claim that may be made by its manufacturer, is not guaranteed or endorsed by the publisher.
